# Bacterial microbiome of the nose of healthy dogs and dogs with nasal disease

**DOI:** 10.1371/journal.pone.0176736

**Published:** 2017-05-01

**Authors:** Barbara Tress, Elisabeth S. Dorn, Jan S. Suchodolski, Tariq Nisar, Prajesh Ravindran, Karin Weber, Katrin Hartmann, Bianka S. Schulz

**Affiliations:** 1 Clinic of Small Animal Medicine, LMU Munich, Munich, Germany; 2 Gastrointestinal Laboratory, Department of Small Animal Clinical Sciences, College of Veterinary Medicine and Biomedical Sciences, Texas A&M University, College Station, Texas, United States of America; Wageningen University, NETHERLANDS

## Abstract

The role of bacterial communities in canine nasal disease has not been studied so far using next generation sequencing methods. Sequencing of bacterial 16S rRNA genes has revealed that the canine upper respiratory tract harbors a diverse microbial community; however, changes in the composition of nasal bacterial communities in dogs with nasal disease have not been described so far. Aim of the study was to characterize the nasal microbiome of healthy dogs and compare it to that of dogs with histologically confirmed nasal neoplasia and chronic rhinitis. Nasal swabs were collected from healthy dogs (n = 23), dogs with malignant nasal neoplasia (n = 16), and dogs with chronic rhinitis (n = 8). Bacterial DNA was extracted and sequencing of the bacterial 16S rRNA gene was performed. Data were analyzed using Quantitative Insights Into Microbial Ecology (QIIME). A total of 376 Operational Taxonomic Units out of 26 bacterial phyla were detected. In healthy dogs, *Moraxella* spp. was the most common species, followed by *Phyllobacterium* spp., *Cardiobacteriaceae*, and *Staphylococcus* spp. While *Moraxella* spp. were significantly decreased in diseased compared to healthy dogs (p = 0.005), *Pasteurellaceae* were significantly increased (p = 0.001). Analysis of similarities used on the unweighted UniFrac distance metric (p = 0.027) was significantly different when nasal microbial communities of healthy dogs were compared to those of dogs with nasal disease. The study showed that the canine nasal cavity is inhabited by a highly species-rich bacterial community, and suggests significant differences between the nasal microbiome of healthy dogs and dogs with nasal disease.

## Introduction

A highly diverse community of microorganisms colonizes human and animal bodies. Since next generation sequencing of 16S rRNA genes has been established as a method to characterize these communities, the understanding of interactions between bacteria and their host has considerably improved. For several organ systems, including gastrointestinal tract [1], skin [2], oral cavity [3], vagina [4], and recently nasal cavity [5], the microbiome of healthy dogs has been described. It was shown that in dogs with gastrointestinal disease, including inflammatory bowel disease and acute diarrhea, alterations in the microbiome are associated with the underlying pathology [6, 7].

The role of bacterial communities in the pathophysiology of canine nasal disease is still unclear. In dogs with chronic rhinitis, bacteria have been discussed as primary or secondary pathogens, as in some patients, at least temporarily, clinical signs improve with antibiotic treatment [8]. In dogs with nasal neoplasia, bacteria are thought to be secondary pathogens, that can colonize the nasal mucosa because of reduced mucosal defense mechanisms [9].

In human medicine, several publications have described the nasal microbiome in healthy individuals [10, 11], and other studies investigated alterations of the bacterial population in patients with inflammatory or neoplastic diseases of the upper airways. Chronic rhinosinusitis in humans for example, is characterized by altered microbial composition and greater abundance of *Staphylococcus aureus* compared to healthy individuals [12]. Different bacterial profiles were also detected in patients with laryngeal carcinoma compared to a healthy control group. *Fusobacterium* and *Prevotella* species were more prevalent in the laryngeal area of these patients than in healthy people [13].

In veterinary medicine, several studies have been published using culture or PCR to investigate bacterial populations in the nasal cavity of dogs [14–16]. However, to date there are only few reports evaluating the nasal microbiome in dogs using next generation sequencing. One study focused on the skin microbiome, including the nostrils, in healthy and allergic dogs. This study revealed a lower species richness and a higher abundance of the family *Moraxellaceae* in the nostrils compared to other skin sites within a population of healthy dogs [2]. A recent study investigated the composition of the upper and lower airway microbiota in healthy dogs in relation to the fecal microbiota. This study illustrated rich microbial populations along the different sites of the canine respiratory tract with increasing relative abundance of *Proteobacteria* from the upper to the lower airways. Furthermore, analysis of the metabolic capacity of canine airway microbiota revealed that respiratory microbes possess the genetic capacity to utilize glyoxylate and citrate cycle metabolic pathways, which allows them to colonize nutrient-poor environments as the airways [5].

So far, there are no studies comparing the nasal microbiome of healthy dogs and dogs with nasal diseases. It is still unknown, if different microbiota possibly represent etiological agents in canine nasal disease. More detailed knowledge about bacterial populations in the dog´s nose might help to understand the question, if microbial changes are primarily leading to a certain disease condition, or if microbial alterations develop secondary to reduced mucosal defense mechanisms, caused by the underlying disease. For the future, knowledge in this field might facilitate new treatment options, including the possibility to support beneficial bacterial groups instead of using antibiotics to repress bacteria colonizing the canine airways. Therefore, the aim of this study was to characterize the nasal microbiome of healthy dogs and compare it to the microbiome of dogs with nasal neoplasia and chronic rhinitis.

## Material and methods

### Ethics statement

The study was approved by the ethics committee of the Centre for Clinical Veterinary Medicine, Faculty of Veterinary Medicine, LMU Munich (number 25-30-04-2014).

### Study population

#### Healthy dogs

Twenty-three healthy dogs (median age 6.0 years, median body weight 15.4 kg) were included in the study (Table 1). All dogs were privately owned and had outdoor access. A history and physical examination were performed in each dog. For inclusion into the study, the dogs had to be without clinical and historical findings suggesting respiratory disease for at least three months prior to sample collection. In addition, dogs were only included if they had not been treated with antibiotic, anti-inflammatory or immunosuppressive drugs for at least four weeks prior to sampling. They had not received any intranasal vaccination during the last three months.

**Table 1:** Study population: Signalement and number of dogs per household in healthy dogs.

Dog	breed	age	sex	weight (kg)	Cephalic index	number of dogs per household
**H1**	Mixed breed	1Y	FS	7.5	mes	1
**H2**	Mixed breed	2Y	FS	14.8	mes	1
**H3**	Beagle	10Y	FS	15.4	mes	4^A^
**H4**	Australian Shepherd	2Y	FS	21.0	mes	4^A^
**H5**	Weimaraner	6Y	FS	22.6	mes	1
**H6**	Mixed breed	4Y	FS	15.0	mes	1
**H7**	Mixed breed	11Y	M	9.5	mes	1
**H8**	German Shepherd	8Y	MN	39.0	dol	1
**H9**	Catahoula Leopard Dog	8Y	M	32.0	mes	2^B^
**H10**	Catahoula Leopard Dog	4Y	FS	28.0	mes	2^B^
**H11**	Mixed breed	6Y	M	25.8	mes	1
**H12**	Portuguese Podengo	8Y	FS	6.9	dol	4^A^
**H13**	Cocker Spaniel	7Y	MN	16.5	mes	2^C^
**H14**	Mixed breed	4Y	F	35.0	dol	2^C^
**H15**	Golden Retriever	7Y	FS	27.0	mes	4^A^
**H16**	Mixed breed	6Y	FS	6.0	mes	4^D^
**H17**	Mixed breed	1Y	M	7.0	mes	4^D^
**H18**	Papillon	3Y	M	5.0	mes	4^D^
**H19**	Mixed breed	8Y	MN	12.0	mes	4^D^
**H20**	Mixed breed	10Mo	F	18.0	mes	1
**H21**	Mixed breed	11Y	MN	10.0	dol	2^E^
**H22**	Dachshund	3Y	F	7.0	dol	2^E^
**H23**	Mixed breed	3Y	FS	20.0	mes	1

Y: years, Mo: months, M: male, MN: male neutered, F: female, FS: female spayed, mes: mesocephalic, dol: dolichocephalic,

^A-E^: dogs living together in the same household marked by the same letter

#### Dogs with nasal disease: Nasal neoplasia

Sixteen dogs with neoplasia of the nasal cavity (median age 9.0 years, median body weight 21.5 kg) were included in the study (Table 2). Malignant nasal neoplasia was diagnosed by histopathology of nasal biopsies. All dogs were client-owned and had outdoor access. Eleven dogs had not been treated with antibiotics at least within the last two weeks prior sample collection, five were receiving antibiotics at the time of sampling. Treatment with anti-inflammatory drugs was no exclusion criterion.

**Table 2:** Study population: Signalement, underlying disease and medication of dogs with nasal neoplasia.

dog	breed	age	sex	weight (kg)	histopathology	antibiotics	anti-inflammatory drugs
**N1**	Golden Retriever	12Y	MN	37.9	esthesioneuroblastoma	no	no
**N2**	Mixed breed	11Y	M	19.5	carcinoma	no	no
**N3**	Husky	2Y	M	19.4	esthesioneuroblastoma	no	no
**N4**	Saint Bernard	8Y	M	61.0	squamous cell carcinoma	no	no
**N5**	Mixed breed	8Y	FS	23.0	carcinoma	no	prednisolone
**N6**	Mixed breed	11Y	FS	6.9	carcinoma	no	no
**N7**	Labrador Retriever	12Y	M	35.3	squamous cell carcinoma	no	no
**N8**	Labrador Retriever	6Y	FS	32.0	lymphoma	no	prednisolone
**N9**	Mixed breed	11Y	F	30.0	osteosarcoma	no	no
**N10**	Coton de Tulear	14Y	M	8.8	carcinoma	no	meloxicam
**N11**	Chihuahua	9Y	M	2.5	carcinoma	no	no
**N12**	Mixed breed	9Y	FS	32.5	carcinoma	amoxi/clav	prednisolone
**N13**	Mixed breed	3Y	FS	20.0	osteosarcoma	amoxi/clav	no
**N14**	Mixed breed	13Y	F	13.0	osteosarcoma	amoxi/clav	prednisolone, metamizole
**N15**	Mixed breed	7Y	MN	37.4	transitional cell carcinoma	enrofloxacin	prednisolone, meloxicam
**N16**	Welsh Corgi	9Y	M	15.0	carcinoma	clindamycin	meloxicam

Y: years, Mo: months, M: male, MN: male neutered, F: female, FS: female spayed

#### Dogs with nasal disease: Chronic rhinitis

Eight dogs with chronic rhinitis (median age 5.0 years, median body weight 19.9 kg) were included (Table 3). Histopathology of nasal tissue in these dogs revealed lymphoplasmacytic (n = 3) or neutrophilic (n = 5) inflammation. Only dogs were included that had no clinical, histological, or cultural evidence of other nasal diseases such as neoplasia, foreign body, or fungal infection. All dogs were client-owned and had outdoor access. Seven of the dogs had not been treated with antibiotics at least within two weeks prior to sample collection, one dog was receiving antibiotics at the time of sampling. Treatment with anti-inflammatory drugs was no exclusion criterion.

**Table 3:** Study population: Signalement, underlying disease and medication of dogs with chronic rhinitis.

dog	breed	age	sex	weight (kg)	histopathology	antibiotics	anti-inflammatory drugs
**CR1**	Old English Sheepdog	3Y	M	43.9	purulent	no	no
**CR2**	Mixed breed	13Y	FS	7.2	purulent	no	no
**CR3**	Mixed breed	6Y	F	12.1	ulcerous, necrotizing, granulomatous	no	no
**CR4**	West Highland White Terrier	2Y	FS	6.6	purulent	no	no
**CR5**	Prager Rattler	5Mo	F	2.3	purulent	no	no
**CR6**	Rhodesian Ridgeback	4Y	MN	44.0	purulent	no	no
**CR7**	Golden Retriever	8Y	M	29.5	lymphoplasmacytic, granulomatous	no	no
**CR8**	Golden Retriever	8Y	F	27.7	lymphoplasmacytic, eosinophilic	clindamycin, doxycycline	no

Y: years, Mo: months, M: male, MN: male neutered, F: female, FS: female spayed

### Sample collection

Two nasal swabs were collected from each dog. For that purpose, a sterile dry rayon swab (Copan^®^ sterile dry swab 155C, Brescia, Italy) was inserted into each nostril and rotated carefully. In the population of healthy dogs, sample collection was performed while the dogs were awake. In the diseased dogs, samples were collected while patients were under general anesthesia before the rhinoscopy procedure was started. All swabs were frozen at -80°C until further analysis.

### DNA extraction

Extraction of the genomic DNA was performed from pooled sets of swabs collected from each dog using a QIAamp^®^ DNA Mini Kit (Qiagen, Hilden, Germany) as recommended by the manufacturer and described previously [17].

Briefly, for lysis of bacteria, swabs were placed in 2 ml phosphate-buffered saline (PBS) containing 0.1% NaN_3_, and were incubated at room temperature for three hours. Swabs were removed and the buffer solution was centrifuged at 7500 rpm for ten minutes (using an Eppendorf Centrifuge 5417R, Hamburg, Germany). After removal of the supernatant and resuspension in 180 μl ATL buffer, the pellet was transferred into a tube with 20 μl proteinase K and incubated at 56°C and 700rpm for one hour (using an Eppendorf Thermomixer Comfort, Hamburg, Germany). Then, 200 μl AL buffer were added and samples were incubated at 70°C for ten minutes. After adding 200 μl of ethanol, samples were transferred into QIAmp Mini spin columns to bind the bacterial DNA. DNA from the two swabs per animal was pooled during this step of the extraction procedure. Afterwards, DNA was washed in two steps following the manufacturer´s instructions. To elute the DNA, 100 μl AE buffer were added on the filter inside the microcentrifuge tube, which then were incubated at room temperature for five minutes and centrifuged at 8000 rpm for one minute. Extracted DNA was frozen at -80°C until further analysis.

### Sequencing

Sequencing of the 16S rRNA gene V4 variable region was performed at MR DNA (www.mrdnalab.com, Shallowater, TX, USA) on an Illumina MiSeq platform following the manufacturer’s guidelines, using forward and reverse primers: 515F (5´-GTGCCAGCMGCCGCGGTAA-3´) and 806R (5´-GGACTACVSGGGTATCTAAT-3´), as described previously [18].

After sequencing, primers and barcodes were removed from the sequences, short, ambiguous, homopolymeric and chimeric sequences were depleted from the dataset using the QIIME (Quantitative Insights Into Microbial Ecology) v1.8 pipeline [19]. Operational Taxonomic Units (OTUs) were assigned based on at least 97% sequence identity using QIIME. The sequences have been deposited in the NCBI Sequence Read Archive under the accession number SRP092120.

### Data analysis

A total of 4,088,256 sequences was amplified throughout all the samples from healthy and diseased dogs. Minimum were 43,193 sequences in one sample, maximum were 135,315 sequences, with a mean of 83,433 sequences (median 83,526). To account for unequal sequencing depth, subsequent analysis was performed on a subset of 43,193 sequences per sample, which is the lowest depth within the samples.

The compiled data were used to determine the relative percentages of bacteria for each individual sample. Alpha and beta diversity measures were calculated and Principle Coordinates Analysis (PCoA) plots and rarefaction curves were generated using the software QIIME v1.8 (Knight and Caporaso Labs, Arizona, USA).

Alpha diversity, a measurement for the diversity of an individual sample, can be described by the number of observed species, the Shannon diversity index, which takes into account abundance and evenness of species [2], and the Chao1 index, which calculates the estimated true species diversity of a sample [20]. To evaluate the beta diversity, a measurement for differences in microbial compositions between different samples, both the weighted UniFrac analysis, which accounts for relative abundance of sequences in different environments, and unweighted, which does not account for relative abundance, were performed.

PCoA plots were investigated for clustering by visual assessment. Factors that were taken into consideration were individual (sex, breed, age, body weight and cephalic index, classified in mesocephalic or dolichocephalic, based on breed or phenotypically suspected breed) and environmental (number of dogs per household) characteristics in the healthy dogs (Table 1), and individual factors (sex, breed, age, body weight, pretreatment with antibiotics, prednisolone, NSAIDs, histopathological diagnosis) in the diseased dogs (Tables 2 and 3). Differences in bacterial communities between healthy dogs and dogs with nasal disease were analyzed using the phylogeny-based unweighted UniFrac distance metric. This analysis measures the phylogenetic distance among bacterial communities in a phylogenetic tree, and thereby provides a measure of similarity among microbial communities present in different biological samples [21]. ANOSIM (Analysis of Similarity) within the software package PRIMER 6 (PRIMER-E Ltd., Luton, UK) was used on the unweighted UniFrac distance matrix to determine significant differences in microbial communities between the different groups. P values <0.05 were considered statistically significant. To elucidate whether dogs, which were living together in the same household, had closer similarities within their microbial communities, average distances between individual animals were calculated using the unweighted UniFrac file using the method ANOSIM with 999 permutations. Thus, the distances between individuals living together were compared to those of dogs living separately.

Statistical analysis of individual factors (age, body weight) and alpha diversity indices were performed using the software package PRISM (PRISM 6, GraphPad Software Inc., San Diego, USA). To avoid influence of confounding factors, dogs with antibiotic pretreatment were excluded from statistical analysis for investigations other than the comparison of pretreated and untreated patients. As in other species, like pigs, an influence of age on the nasal microbiota has been shown [22], dogs under 12 months of age were also excluded from statistical analysis except age-related statistics.

Because the data were assumed to be not normally distributed, a non-parametric Mann-Whitney test was used for statistical comparison between healthy and diseased dogs. A non-parametric Kruskal-Wallis test followed by Dunn`s Multiple Comparison post-test was performed to compare individual factors and alpha indices for the three groups healthy, nasal neoplasia, and chronic rhinitis. To determine which disease types were significantly different with regard to these factors, an additional pairwise test using PRIMER 6 was used. P values <0.05 were considered statistically significant.

Linear discriminant analysis effect size (LEfSe) was used to elucidate bacterial taxa (16S rRNA genes) associated with healthy or diseased dogs. LEfSe was used online in the Galaxy workflow framework (https://huttenhower.sph.harvard.edu/galaxy/).

Differences in the proportions of bacterial taxa between healthy and diseased dogs were investigated using a non-parametric Kruskal-Wallis test, using the statistical package JMP Pro 11 (SAS, Marlow, Buckinghamshire). Resulting p-values were corrected for multiple comparisons using the Benjamini & Hochberg False Discovery Rate [23]. A Dunn’s Multiple Comparisons post-test was used to determine which disease types were significantly different.

## Results

### Animal population

A significant difference (p = 0.015) was observed concerning age between healthy dogs (5.4 ± 3.2 years) and those with nasal neoplasia (9.1 ± 3.4 years), but not between healthy dogs and dogs with chronic rhinitis (5.6 ± 4.1 years). No significant difference in body weight was identified between healthy dogs (17.5 ± 9.9 kg) and dogs with nasal tumors (24.6 ± 14.8 kg) or chronic rhinitis (21.7 ± 16.9 kg).

### Nasal microbiome of healthy dogs

#### Nasal microbial composition

Investigating PCoA plots, no clustering, based on similarities of bacterial molecular phylogenetic trees, was observed when comparing sex, age group (< 1 year, 1–3 years, 4–8 years, 9–12 years, > 12 years), body weight group (< 10 kg, 10–20 kg, > 20 kg) or cephalic index between groups. This was confirmed with statistical testing using ANOSIM on the unweighted UniFrac distance metric, which showed no significant differences for these comparisons (sex: p = 0.151, R = 0.012, age group: p = 0.320, R = 0.035, body weight group: p = 0.05, R = 0.121, cephalic index: p = 0.739, R = -0.091).

Calculation of average distances using the unweighted UniFrac distance metric showed no closer similarity of microbial communities between dogs living together in one household when compared to dogs living separately.

#### Species richness and diversity

**Fig 1:**
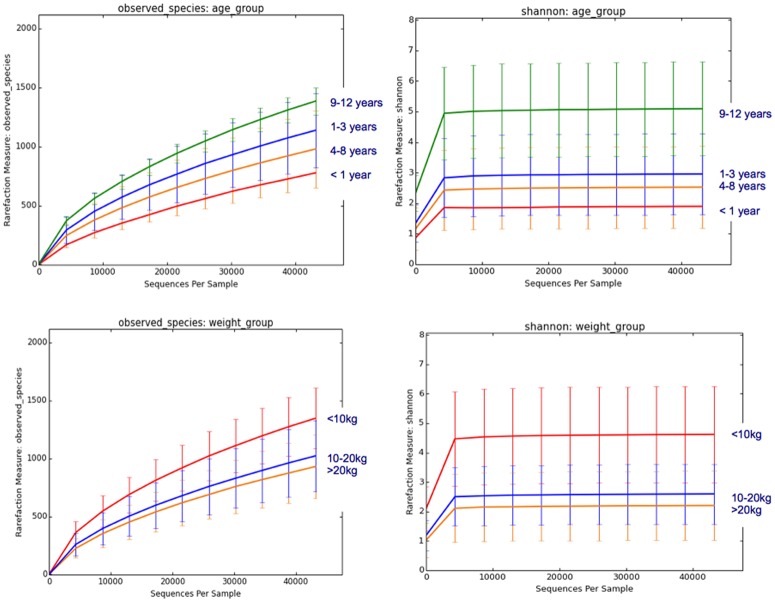
Rarefaction analysis of 16S-rRNA gene sequences obtained from healthy dogs, comparing different age and body weight groups. Lines represent the mean and error bars represent standard deviations. Shannon diversity index and number of observed species are higher in dogs older than 9 years and in dogs with a body weight of less than 10kg.

A rarefaction analysis was performed to evaluate species richness. For different age groups and different weight groups significant differences between single groups could be demonstrated (Fig 1). Dogs older than 9 years (age group 4) had a significantly higher Shannon diversity index than younger dogs (age group 3, 4–8 years old) (ANOVA with following Tukey test, p = 0.036). Differences in number of observed species and Chao1 were not significant. Considering different weight groups, dogs with a body weight of less than 10 kg had a significantly higher Shannon diversity index (p = 0.017) and number of observed species (p = 0.041) than dogs with a body weight over 10 kg. Chao1 did not differ significantly. Other factors like breed or time of the year, when the animal was sampled, were too variable to be evaluated statistically.

Good´s coverage was higher than 0.97 in all samples at the chosen sequencing depth of 43,190 sequences. This allows to conclude that all the samples have a sufficiently high number of sequences and enough coverage, and all animals were equally sampled.

#### Most common taxa colonizing the nasal cavity of healthy dogs

A total of 26 bacterial phyla was detected in the samples from the nasal cavity of healthy dogs. Most abundant phylum was *Proteobacteria* (mean 83.4%, min 37.4%—max 98.5%), followed by *Firmicutes* (4.8%, 0.4–20.8%), *Bacteroidetes* (2.6%, 0.1–12.5%), *Cyanobacteria* (2.1%, 0.0–11.6%), and *Actinobacteria* (2.1%, 0.1–8.6%) (Table 4). Other phyla, like *Verrucomicrobia*, *Tenericutes*, *Planctomycetes* and GN02 were detected in smaller amounts and only in a few animals.

**Table 4:** Taxa present at >1% mean relative abundance in healthy and diseased dogs. Mean relative percentages and standard deviation of the most abundant bacterial groups, annotated to the level of phylum, family and Operational Taxonomic Unit (OTU), based on sequencing of the 16S rRNA.

Taxon	healthy		neoplasia		rhinitis			
phylum family *OTU*	mean* %	SD %	mean %	SD %	mean %	SD %	Kruskal Wallis p-value	q-value**
**Proteobacteria**	82.8	14.8	72.4	29.3	64.3	20.4	0.133	0.448
Moraxellaceae	60.5^a^	30.4	22.8^b^	29.0	40.9^a^^b^	32.7	**0.006**	0.323
*Moraxella spp*.	58.0 ^a^	30.2	14.9 ^b^	21.5	33.8 ^a^^b^	31.5	**0.001**	0.188
Phyllobacteriaceae	3.5	14.7	0.1	0.0	0.1	0.1	0.239	0.690
*Phyllobacterium spp*.	3.5	14.7	0.1	0.0	0.1	0.0	0.573	0.813
Neisseriaceae	2.7	5.8	15.5	18.4	3.1	3.3	0.077	0.545
*Conchiformibius spp*.	0.9	1.5	9.5	15.9	1.8	2.2	0.429	0.740
Cardiobacteriaceae	2.3	3.6	0.4	0.6	1.2	2.5	0.039	0.457
Polyangiaceae	1.4	6.6	0.0	0.0	0.0	0.0	0.051	0.457
Comamonadaceae	1.3	2.6	1.4	2.0	0.9	1.0	0.927	0.991
Pasteurellaceae	0.7 ^a^	0.8	15.6 ^b^	20.1	3.4 ^a^^b^	4.1	**0.019**	0.457
*Pasteurella multocida*	0.1 ^a^	0.1	2.5 ^b^	6.5	0.0 ^a^^b^	0.0	**0.004**	0.209
Xanthomonadaceae	0.2	0.2	3.3	6.9	0.4	0.3	0.206	0.669
Pseudomonadaceae	0.4	0.8	2.0	3.5	1.1	1.5	0.339	0.723
Alcaligenaceae	0.5	0.7	1.0	1.4	1.4	1.7	0.601	0.833
Enterobacteriacae	0.2	0.3	1.0	2.5	0.4	0.4	0.392	0.747
Sphingomonadaceae	0.8	0.7	0.3	0.5	2.4	4.5	0.119	0.597
Oxalobacteraceae	0.3	0.5	0.5	0.9	1.6	2.9	0.661	0.881
Acetobacteraceae	0.1 ^a^	0.2	0.0 ^b^	0.0	1.1 ^a^^b^	2.1	**0.041**	0.457
**Firmicutes**	4.9	6.2	7.2	8.7	4.7	3.4	0.359	0.554
Staphylococcaceae	1.8	4.4	1.8	2.9	1.6	1.7	0.816	0.963
[order] Clostridiales	0.1	0.1	1.5	4.8	0.0	0.0	0.145	0.600
**Bacteroidetes**	2.8	3.3	5.5	7.2	11.6	11.4	0.127	0.448
[Weeksellaceae]	0.6	1.0	1.6	1.7	4.8	10.9	0.246	0.690
Porphyromonadaceae	0.2	0.6	1.1	3.6	0.4	0.5	0.296	0.723
Chitinophagaceae	0.9	1.6	1.1	1.4	4.0	6.7	0.540	0.826
Cytophagaceae	0.7	0.9	0.3	0.4	2.2	2.2	0.222	0.672
**Cyanobacteria**	2.1	3.4	0.6	1.0	7.1	7.7	0.315	0.531
[order] Streptophyta	1.7	3.3	0.5	1.0	6.4	7.2	0.303	0.723
**Actinobacteria**	2.1	2.1	6.0	10.8	3.8	3.8	0.750	0.843
Microbacteriaceae	0.5	0.6	1.3	3.7	0.3	0.3	0.192	0.669
*Leucobacter spp*.	0.3	0.4	1.2	3.7	0.1	0.1	0.097	0.602
Micrococcaceae	0.3	0.6	3.8	10.3	0.4	0.6	0.262	0.716
**GN02**	1.9	3.0	1.3	1.6	0.7	1.1	0.253	0.531
[class] BD1-5	1.9	3.0	1.3	1.6	0.7	1.1	0.204	0.669
**Spirochaetes**	0.0	0.0	1.5	4.7	0.0	0.0	0.130	0.448
Spirochaetaceae	0.0	0.0	1.3	4.4	0.0	0.0	0.052	0.457
*Treponema spp*.	0.0	0.0	1.3	4.4	0.0	0.0	0.052	0.598
**Tenericutes**	0.1	0.2	2.0	5.5	0.9	1.6	0.395	0.554
Mycoplasmataceae	0.1	0.2	1.9	5.5	0.9	1.6	0.840	0.964
*Mycoplasma spp*.	0.1	0.2	1.9	5.5	0.9	1.6	0.856	0.915

*^a, b^: Means not sharing a common superscript differ significantly (p < 0.05, Dunn's multiple comparison test).

** q‐values adjusted based on the Benjamini & Hochberg False discovery rate

**Fig 2:**
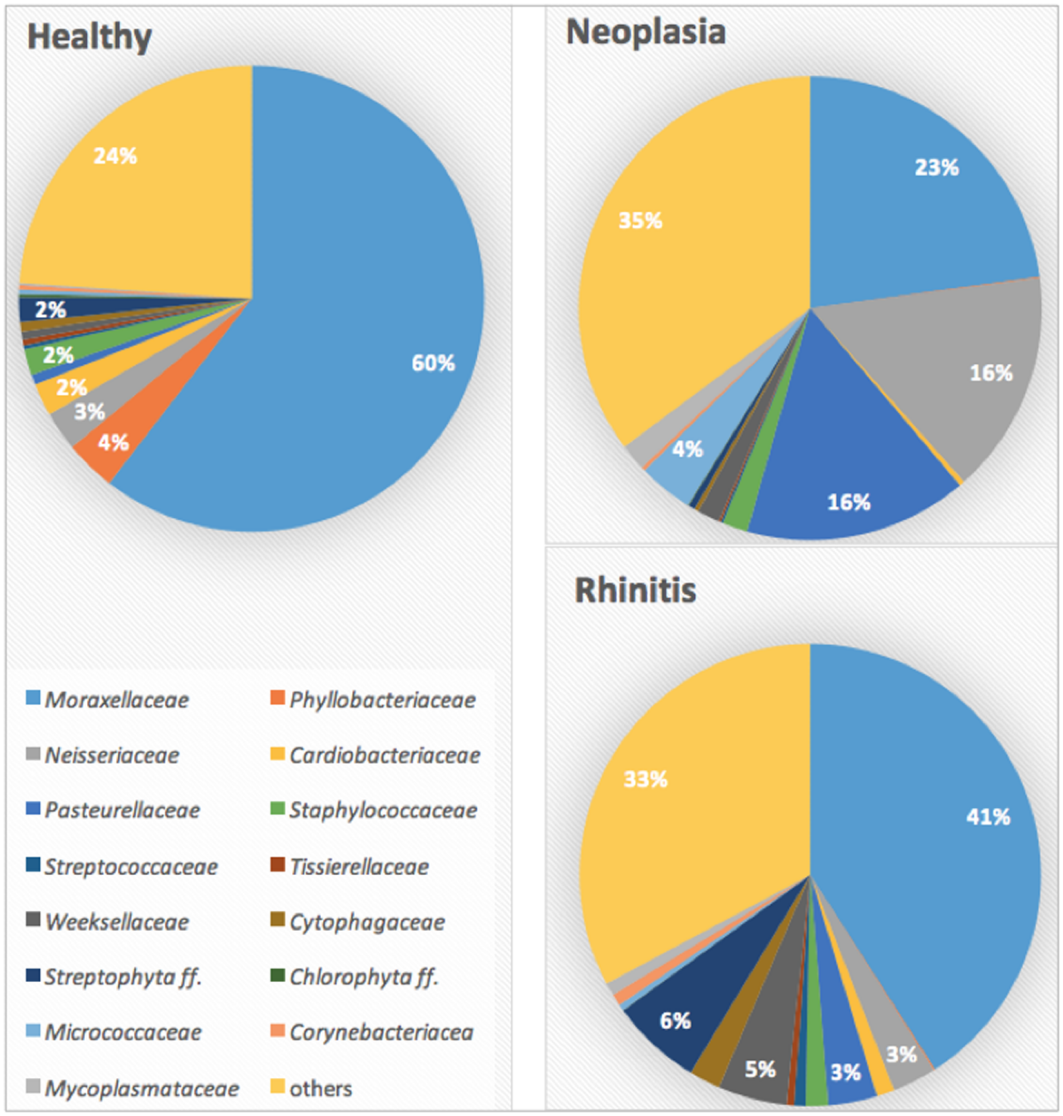
Bacterial families in healthy and diseased dogs. Mean values of most common bacterial families in the nasal cavity of healthy dogs, dogs with nasal neoplasia and chronic rhinitis.

At class level, *Gammaproteobacteria* were most commonly detected, followed by *Alphaproteobacteria*, *Betaproteobacteria*, and *Bacilli*. Most frequently detected family was *Moraxellaceae* (phylum *Proteobacteria*, class *Gammaproteobacteria*, order *Pseudomonadales*) (Fig 2). *Moraxella* was the genus which was identified predominantly in most of the samples of healthy dogs. This genus was detected in all samples, and represented 59.2% of the total taxa in healthy dogs, with a range of 1.5% to 95.6%. Other frequently identified genera were *Phyllobacterium* (3.4%, 0.1–12.8%), not specified genera of the family *Cardiobacteriaceae* (2.1%, 0.0–69.1%), and *Staphylococcus* (1.7%, 0.0–15.3%); however, these genera were predominantly represented only in few individuals.

Throughout all samples, 376 different OTUs were detected, but DNA of most of the highly abundant taxa could not be resolved beyond genus level.

### Nasal microbiome of healthy dogs compared to diseased dogs

#### Nasal microbial communities in healthy versus diseased dogs

**Fig 3:**
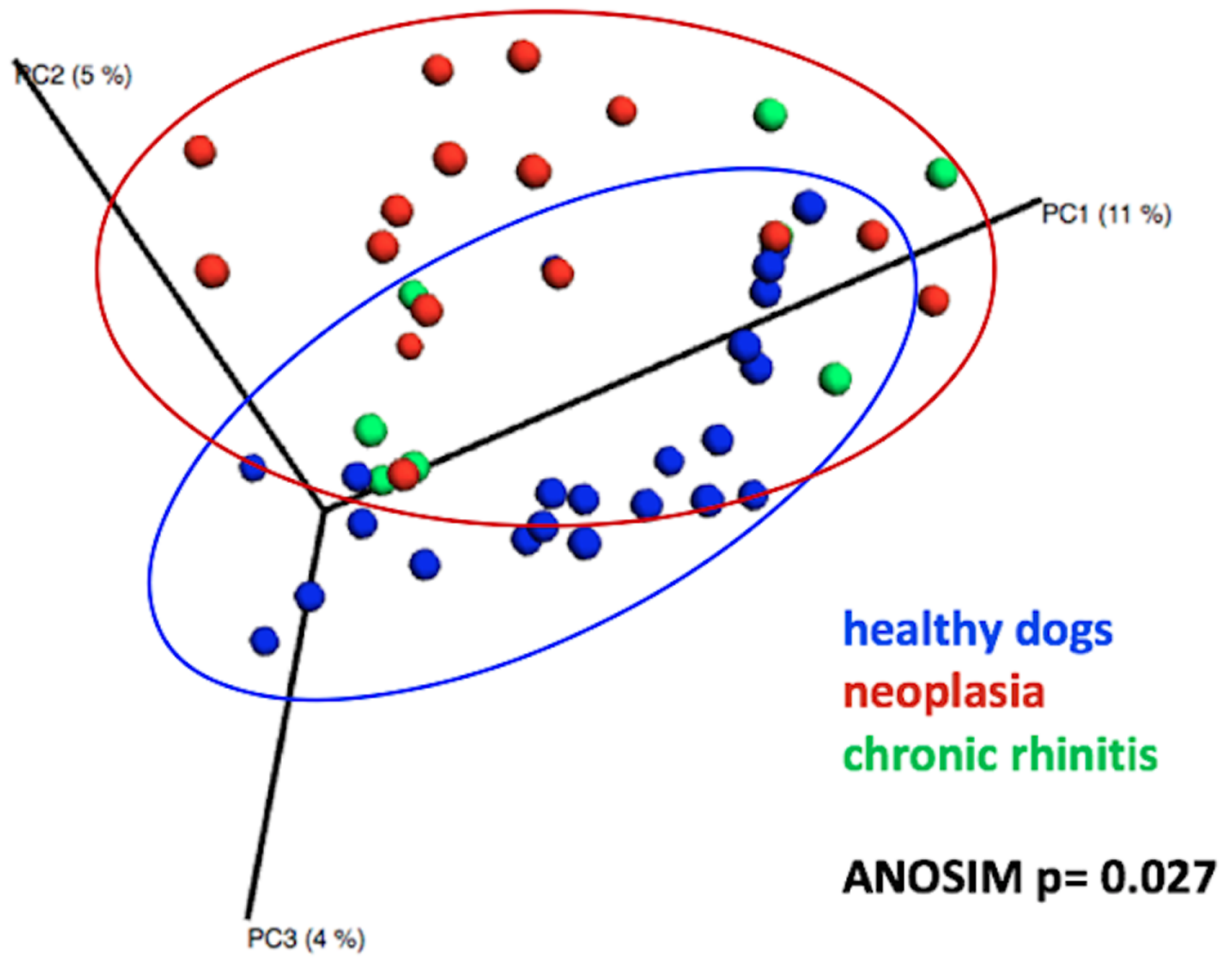
Principal coordinate analysis (PCoA) of unweighted UniFrac distances of 16S rRNA genes (3D). Similarities in microbial communities in healthy dogs, dogs with nasal neoplasia and chronic rhinitis. Clustering is observed between healthy dogs and dogs with nasal neoplasia, but not in dogs with chronic rhinitis.

Clustering in the PCoA plots between microbial communities of healthy and diseased dogs was observed. While healthy dogs formed a cluster, and dogs with nasal neoplasia formed another cluster, microbial communities in dogs with chronic rhinitis were more scattered (Fig 3). In ANOSIM analysis a significant difference could be verified when comparing healthy and diseased dogs (p = 0.027, R = 0.101). Pairwise test using PRIMER6 suggested that healthy dogs and dogs with nasal neoplasia differed significantly (p = 0.033) in microbial community composition. Between healthy dogs versus dogs with chronic rhinitis (p = 0.590) and dogs with chronic rhinitis versus dogs with nasal neoplasia (p = 0.390) no significant difference was verified.

Most of the individual factors (sex, breed, age, body weight, pretreatment with antibiotics, prednisolone, NSAIDs, histopathological diagnosis) were highly variable. Due to the resulting small sample size, statistical comparison of male and female dogs, different age groups, different groups of body weight, and patients with different histological diagnoses within the group of either neoplasia or chronic rhinitis, was not performed. There was no clustering observed, assessing the PCoA plots of microbial communities, with exception of antibiotic treatment within the group of dogs with nasal neoplasia.

**Fig 4:**
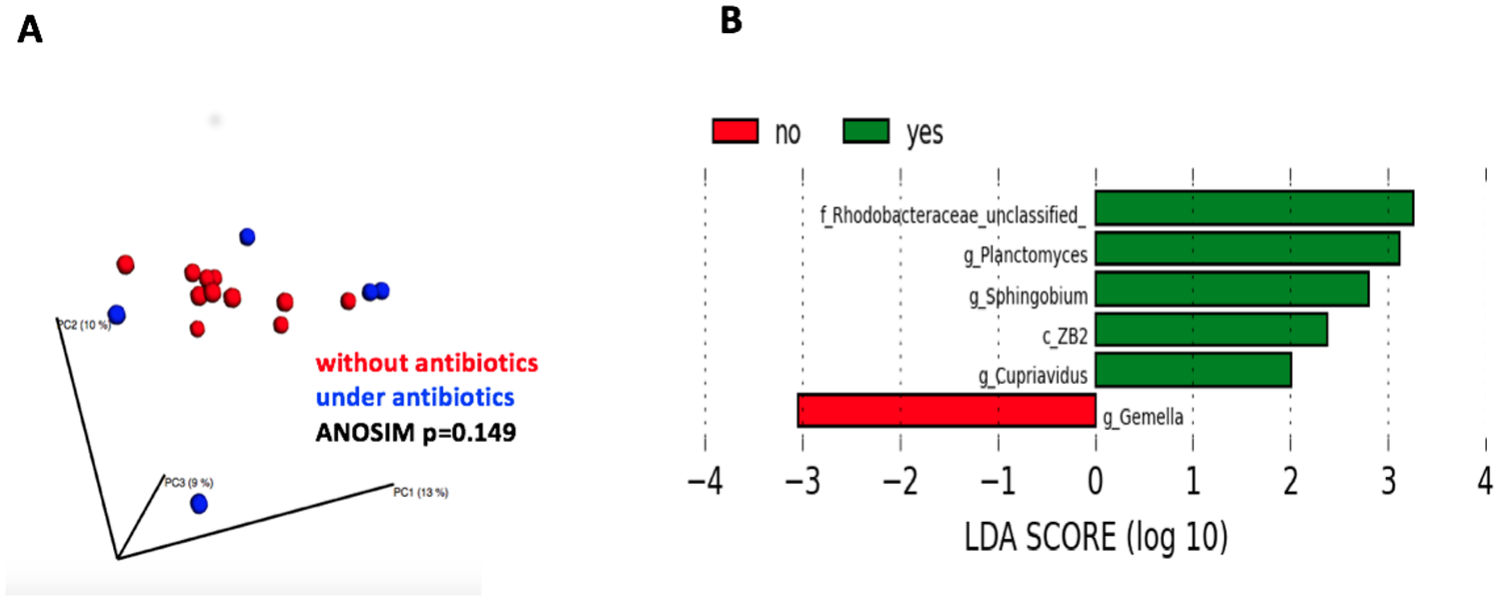
Differences in microbial communities in dogs with nasal neoplasia depending on antibiotic treatment. PCoA plots of microbial communities in dogs with nasal neoplasia without and under antibiotic treatment **(A)**, bacterial taxa significantly higher represented in dogs with (green) and without (red) antibiotic treatment showed by linear discriminant analysis (LDA) scores based on LEfSe **(B)**.

Within the group of dogs with nasal neoplasia, bacterial communities of patients with and without antibiotic treatment were compared statistically. The microbial communities of pretreated dogs were marginally distributed compared to untreated dogs (Fig 4A), but this was not significant based on ANOSIM of unweighted UniFrac distance metrics (p = 0.149, R = 0.136). Although there was no significant difference demonstrated for ß-diversity, several significantly different bacterial taxa could be detected using LEfSe (Fig 4B). While the genera *Planctomyces* and *Sphingobium* and the family *Parachlamydiaceae* were significantly more represented in dogs that had received antibiotics within the last two weeks, the family *Gemellaceae* was associated with dogs without antibiotic treatment.

#### Species richness and diversity

**Fig 5:**
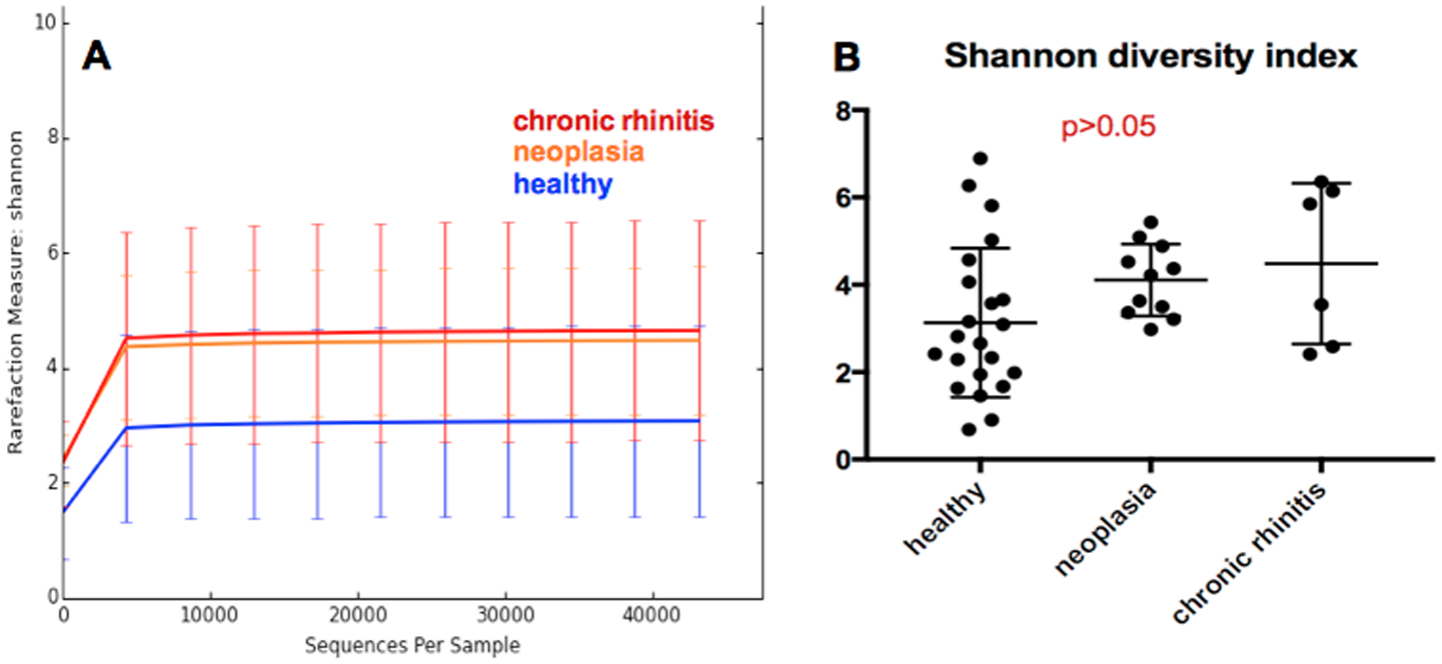
Rarefaction curve and statistical evaluation of Shannon diversity index. Shannon diversity index is lower in healthy dogs (blue) than in dogs with chronic rhinitis (red) or nasal neoplasia (orange) (dogs with antibiotic pre-treatment excluded) **(A)**. The difference is not significant in a Dunn´s multiple comparison test **(B)**.

Diversity analysis was performed to compare the number of observed species, Shannon diversity index, Chao1 between healthy and diseased dogs. Statistical analysis of these parameters showed no significant differences between the groups for number of observed species and Chao1. Shannon diversity index was lower for the healthy dogs than for the diseased dogs (Kruskal-Wallis test, p = 0.038), but the difference was not statistically significant when comparing the three groups, healthy dogs versus nasal neoplasia versus chronic rhinitis, in the following Dunn´s multiple comparison test (Fig 5).

#### Most common taxa colonizing the nasal cavity of healthy compared to diseased dogs

**Fig 6:**
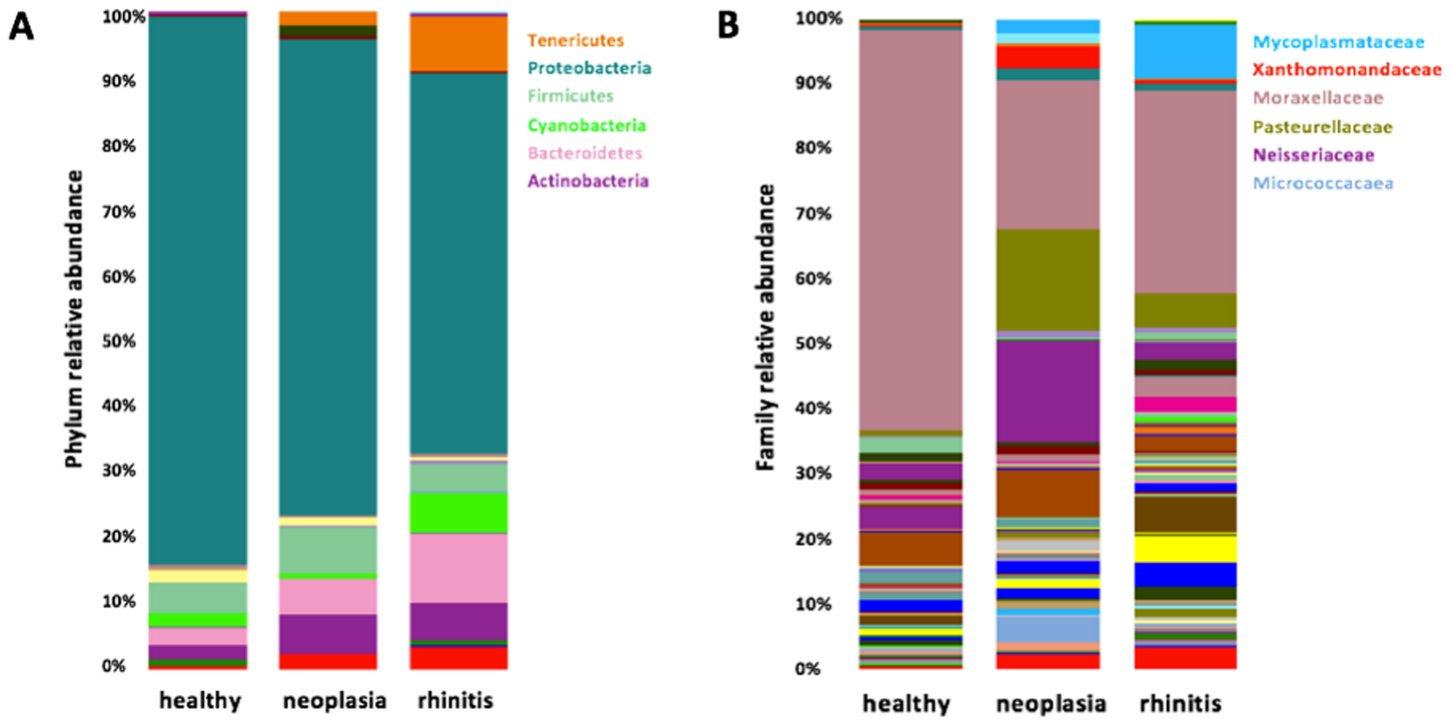
Composition of the nasal microbiome in healthy and diseased dogs. Bar charts showing relative abundance of all taxa detected in nasal swabs of dogs, annotated to the taxonomic level of phylum **(A)** and family **(B)**.

In dogs with nasal neoplasia, *Moraxella* spp. was the most abundant finding on genus level, as it was in healthy dogs, but accounted for only 15.3% of the total taxa (min. 0.7%–max. 61.1%). This difference was statistically significant (p = 0.001). The proportions of the family *Pasteurellaceae* (phylum *Proteobacteria*, class *Gammaproteobacteria*, order *Pasteurellales*) were different between healthy and diseased dogs. This bacterial family accounted for 12.9% (0.1–64.3%) of the total taxa and was significantly higher represented in dogs with neoplasia (p = 0.019) (Figs 2 and 6). *Haemophilus parainfluenza* (p = 0.018) and *Pasteurella multocida* (p = 0.004) were representatives of this family that were significantly more common in dogs with nasal neoplasia than in healthy dogs. Other common genera in dogs with nasal neoplasia included *Conchiformibius* spp. (9.5%, 0.1–49.9%, p = 0.429) (phylum *Proteobacteria*, family *Neisseriaceae*) and non-specified genera of the families *Neisseriaceae* (5.9%, 0.1–54.5%, p = 0.080) and *Micrococcaceae* (3.2%, 0.1–34.8%, p = 0.480); however, these were not more commonly detected than in healthy dogs.

**Fig 7:**
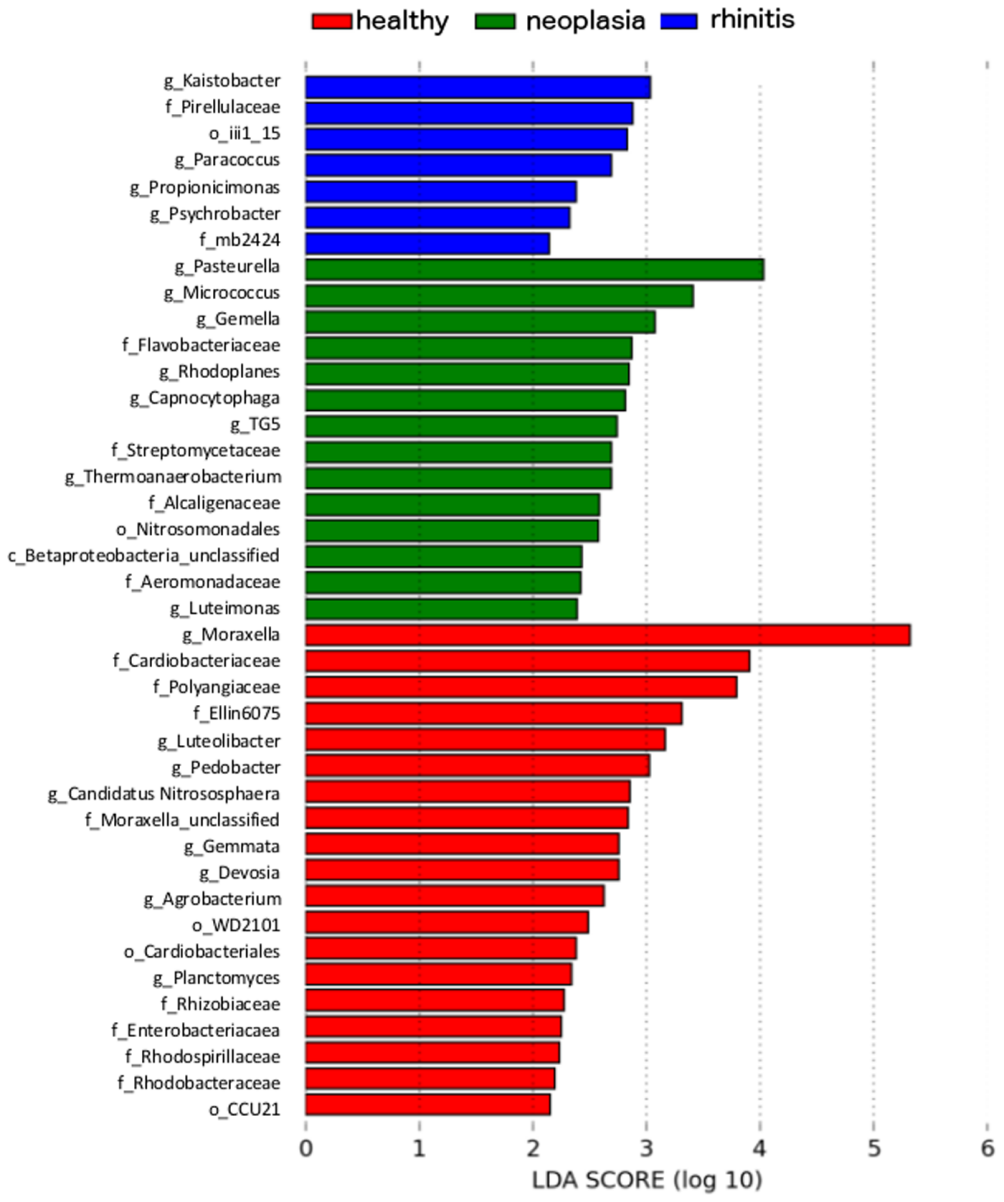
Different relative abundance of bacterial taxa between healthy and diseased dogs. Bacterial taxa at different taxonomic levels (c = class, o = order, f = family, g = genus) with significantly different mean relative abundance in dogs with chronic rhinitis (blue), nasal neoplasia (green) and healthy dogs (red), ranked according to their effect size determined by LDA score, based on LEfSe.

Analysis of individual bacterial groups based on LDA effect size (LEfSe) observed alterations in several taxa when dogs with nasal neoplasia were compared to healthy dogs (Fig 7). Dogs with nasal tumors showed significantly decreased relative abundance of *Moraxella* spp. (p = 0.001) and *Cardiobacteriaceae* (p = 0.039), while *Pasteurella* spp. (p = 0.004) was significantly increased.

The nasal cavity of dogs with chronic lymphoplasmacytic or neutrophilic rhinitis was also predominantly colonized by *Moraxella* spp. (34.5%, 0.7–77.3%), followed by the order *Streptophyta* (6.4%, 0.0–16.6%), the genus *Riemerella* spp. (4.4%, 0.0–25.3%), and the family *Pasteurellaceae* (2.9%, 0.2–17.1%) (Figs 2 and 6).

**Fig 8:**
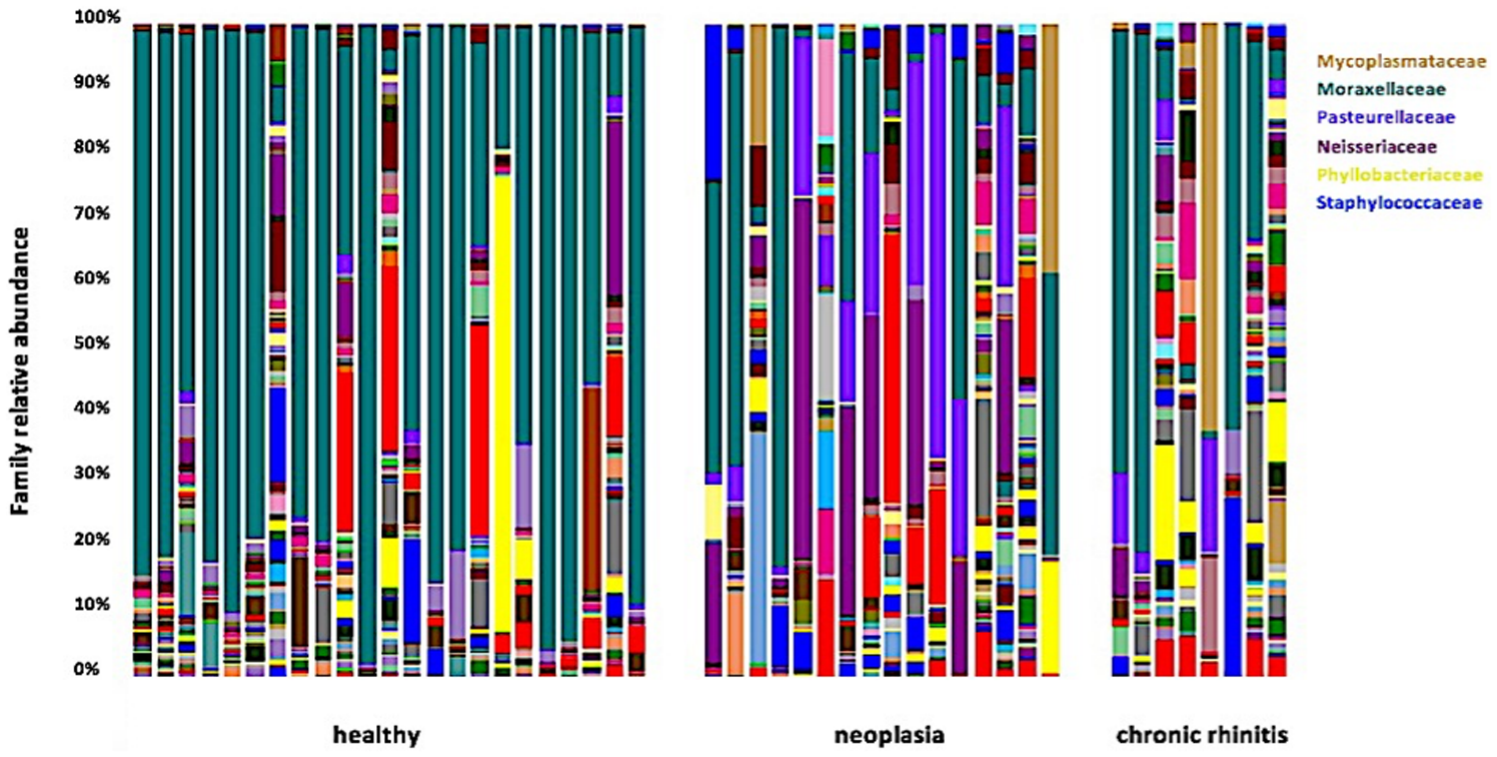
Individual family-level composition of the nasal microbiome in healthy and diseased dogs. Bar charts showing relative abundance of all taxa (annotated to the taxonomic level of family) detected in nasal swabs of dogs. Each bar chart represents one dog.

When individual bacterial groups were analyzed by LEfSe, a significant difference between healthy dogs and dogs with chronic rhinitis could be observed for several taxa, including *Kaistobacter* spp. (0.3%, 0.0–1.1%, p = 0.036) and *Pirellulaceae* (0.2%, 0.0–0.9%, p = 0.049) (Fig 7). However, these accounted for only a small percentage of the total taxa (<0.3%) and only for single animals. *Mycoplasma* spp. were considerably more abundant in several individual dogs with chronic rhinitis, especially in the sample of one young dog with chronic rhinitis (61.8%) (Fig 8), but since dogs under 12 months of age were excluded from other than age-related statistical analysis, this dog was not included in the comparison.

All taxa that differ between groups, are listed in the supporting information (S1 Table).

## Discussion

The study demonstrates that the canine nasal cavity is inhabited by a species-rich bacterial community. In agreement with previous investigations, which used next-generation sequencing methods for bacterial analysis, the nasal microbiome of healthy dogs was shown to be dominated by *Moraxellaceae*, especially *Moraxella* spp., followed by several other bacterial families at considerably lower levels [2, 5]. Possible reasons for a different order of the detected taxa, if sorted based on abundance, could be the selection of study subjects. While in one of the previous studies [5] samples from a uniform cohort of research animals were used, the present study included a heterogeneous group of pet dogs for analysis. The different genetic and environmental background of these populations could be a confounding factor.

Several bacterial taxa isolated in this study have been described for the first time since next-generation sequencing methods complement culture-based methods. These bacteria have never been associated with the dog´s nasal cavity before. Many of these bacteria cannot be cultured so far, because they are highly adapted to their particular microenvironment, which is difficult to reproduce under laboratory conditions [24]. Some have been known as being part of the canine microbiome, such as *Porphyromonas*, GN02, or *Conchiformibius* in the oral cavity [25, 26]. Other taxa have been detected in soil or water samples before [27, 28]; thus, it is likely that these bacteria were obtained from the environment, as the nares are very exposed to a dog´s outdoor environment.

In previous studies, in which culture-based methods were used, a much lower number of bacterial taxa were identified in the canine nose compared to recent investigations using pyrosequencing methods. In healthy dogs, *Staphylococci*, *Streptococci*, *Acinetobacter*, and *Enterococci* were hypothesized to be the main components of the nasal microbiota [14, 29, 30]. Predominant taxa detected using next generation sequencing, such as *Moraxella* spp., did not occur in the results obtained by culturing methods.

In human medicine, there are several studies investigating the nasal microbiome of healthy as well as diseased individuals based on 16S rRNA sequencing. Different microbiomes for different sites in the upper airways have been described [10, 31]. In humans, *Actinobacteria* and *Firmicutes* accounted for the majority of nasal bacteria, with a lower prevalence of *Proteobacteria*. Therefore, there seem to be considerable differences between the nasal bacterial communities of different species.

In veterinary medicine, only few reports exist about microbial colonization of the upper airways in dogs with nasal disease. The present study is the first one to investigate nasal microbiota in dogs with nasal disease using next-generation sequencing methods. Bacteria of the upper respiratory tract of dogs with respiratory signs have been examined using cultural methods before [32]. Most frequently isolated bacteria were *Staphylococcus intermedius*, *E*. *coli*, α-hemolyzing *Streptococcus*, and *Pasteurella multocida*, which were all not considered primary pathogens, but probably originating from the normal bacterial community. With exception of *E*. *coli*, these bacteria could also be found in the present study population, but accounted only for a minor proportion of the microbiota in healthy and diseased animals. While different species of *Staphylococcus* and *Pasteurella* were detected at low levels in most of the animals, *Streptococcus* occurred only in a small number of dogs.

In another study, bacterial cultures of dogs with nasal neoplasia and lymphoplasmacytic rhinitis were examined. An equal distribution between negative culture results and bacteria that were considered normal flora was detected in both groups; however, the definition of “normal microflora” was based on data obtained from the literature [33].

Nasal biopsies from dogs with lymphoplasmacytic rhinitis (LPR), nasal neoplasia, and aspergillosis have been examined for bacterial and fungal DNA using polymerase chain reaction in a previous work [16]. No difference in DNA load for bacterial DNA could be demonstrated between disease groups. Furthermore, no bacterial taxa which have been associated with upper respiratory tract disease in several species, like *Bartonella*, *Mycoplasma*, or *Chlamydia* [34–36], could be detected in any biopsy. However; in the present study facultative pathogens such as *Mycoplasma* spp. were also only detected in very small numbers.

The present study was able to demonstrate alterations of the nasal microbiome of dogs with nasal neoplasia as well as chronic rhinitis compared to healthy dogs. Most noticeable findings were the decreased abundance of *Moraxellaceae* and higher abundance of *Pasteurellaceae* in diseased dogs. Also, the family *Neisseriaceae* was apparently more common in some of the individuals affected by nasal neoplasia (Fig 7), although this difference was not statistically significant for all the dogs. So far it is not possible to elucidate, whether these alterations play a role in the etiology of the disease process or rather represent consequences of a primary disease. One conceivable mechanism could be bacterial overgrowth with certain taxa, enabled by immune modulation caused by the underlying disease. However; some of the bacteria found in the present study are known to be capable to subvert their host´s immune system. In toxigenic strains of *Pasteurella multocida* the protein toxin PMT is found, which acts as a strong mitogen, protects from apoptosis and has an impact on the differentiation and function of immune cells [37]. This could be one possible mechanism to support progression of the disease.

In human medicine, several studies demonstrated a difference in nasal bacterial communities when comparing healthy humans to patients with chronic rhinosinusitis (CRS). CRS patients were characterized by altered microbial composition and greater abundance of *S*. *aureus* [12]. A quantitative increase in most bacterial and fungal species was reported in patients with CRS relative to controls, but qualitatively similar microbiomes [24]. This study also performed a more detailed characterization of the immune response. Flow cytometry was performed to measure contents of immune cells in lavage of the middle meatus in CRS and control patients, and cytokines and chemokines were measured by multiplexed ELISA. Results of this investigations demonstrated significantly elevated T_H_2-related cytokines and increased interleukin (IL) 8 in patients with CRS. Immune response of peripheral blood leukocytes of CRS patients cocultured with lavage of healthy individuals was analyzed measuring the IL-5 secretion (i.e. T_H_2 response) by ELISpot assay. Data supported the theory that in some cases, CRS results from an immune hyperresponsiveness to the commensal microbiome. No similar investigations have been performed in dogs with chronic rhinitis so far, but it would be interesting to investigate the immune response of dogs with chronic rhinitis.

For the development of neoplastic diseases, an involvement of microbiome alterations and dysbiosis has been discussed in different human studies as well [13, 38]. One study reported that the bacterial profiles of the larynx of laryngeal cancer patients were significantly different from those of healthy control subjects. It suggested a potential role of several microorganisms in the pathogenesis of laryngeal carcinoma, for example *Fusobacterium* spp. as proinflammatory pathogens [13]. To what extent the altered bacterial taxa, like increased incidence of *Pasteurellaceae*, found in the dogs with nasal neoplasia in the present study could be involved in cancerogenesis, remains a topic for further research.

An interesting finding was that the nasal microbial composition of dogs with nasal tumors was not significantly altered if dogs had been pretreated with antibiotics. In a study investigating the gastrointestinal microbiota in dogs before and under antibiotic treatment, considerable and prolonged effects on bacterial composition could be demonstrated [39]. However, due to the relatively small number of diseased patients in the present study and the heterogeneous treatment protocols with different antimicrobial and partially anti-inflammatory drugs, validity of this finding is limited. Nevertheless, only a few taxa were altered significantly between pretreated and untreated patients, and the diversity and number of observed species was similar. Possible reasons could be insufficient accumulation of antibacterial drugs in the nasal mucosa, bacterial resistance to antibiotics, or detection of DNA from nonviable bacteria.

Limitations of the present study are the relatively small number of animals per group, and a heterogeneous pretreatment within the population of diseased animals. Furthermore, different settings of sample collection between healthy dogs being awake and diseased dogs being under anesthesia could be a confounding factor leading to different results.

## Conclusion

Using next-generation sequencing methods, a highly species-rich bacterial community was shown to inhabit the canine nasal cavity. The majority of bacteria detected in this study had never or only rarely been isolated before with conventional culture techniques. Significant differences in the composition of microbiota colonizing the nose of healthy dogs compared to dogs with nasal neoplasia or chronic rhinitis suggest a complex role of the nasal microbiome in the disease process. Further studies are warranted to elucidate the complex interactions between nasal microbiome, host immune response, and canine nasal disease.

## Supporting information

S1 Table Mean relative percentages of the bacterial groups differing between healthy and diseased dogs, at various phylogenetic levels (phylum, class, order, family, genus, species), based on sequencing of the 16S-rRNA-gene. (XLSX)
